# Crystal structure of bis­(η^2^-ethyl­ene)(η^5^-penta­methyl­cyclo­penta­dien­yl)cobalt

**DOI:** 10.1107/S2056989016012822

**Published:** 2016-08-16

**Authors:** Chandika D. Ramful, Katherine N. Robertson, Kai E. O. Ylijoki

**Affiliations:** a Department of Chemistry, Saint Mary’s University, 923 Robie St., Halifax, NS, B3H 3C3, Canada

**Keywords:** crystal structure, penta­methyl­cyclo­penta­dien­yl, olefin complex, cobalt

## Abstract

The title compound, [Co(C_10_H_15_)(C_2_H_4_)_2_], was prepared by Na/Hg reduction of [Co_2_(C_10_H_15_)_2_(μ-Cl)_2_] in THF under an ethyl­ene atmosphere and crystallized from pentane at 193 K. The Co—C(olefin) bonds have an average length of 2.022 (2) Å, while the Co—C(penta­dien­yl) bonds average 2.103 (19) Å. The olefin C=C bonds are 1.410 (1) Å. The dihedral angle between the planes defined by the cyclo­penta­dienyl ligand and the two olefin ligands is 0.25 (12)°. In the crystal, mol­ecules are linked into chains by C—H⋯π inter­actions.

## Abstract 2

In the title compound, the Co—C(olefin) bonds have an average length of 2.022 (2) Å, while the Co—C(penta­dien­yl) bonds average 2.103 (19) Å. The olefin C=C bonds are 1.410 (1) Å. In the crystal, mol­ecules are linked into chains by weak C—H⋯π inter­actions.

## Chemical context   

The title compound, Cp*Co(CH_2_CH_2_)_2_ (Cp* = penta­methyl­cyclo­penta­dien­yl), was first reported in 1981 by Spencer and coworkers (Beevor *et al.*, 1981 ▸) in their quest to find a more thermally labile analogue of the related Cp*Co(dicarbon­yl) complex. Since this first report, it and other olefin complexes of cobalt with Cp* or Cp (Cp = cyclo­penta­dien­yl) have become important precursors for the generation of Cp′Co*L* (*L* = olefin, pyridine, *etc*) and Cp′Co fragments used as active species in C—H bond activation (Lenges *et al.*, 1997 ▸, 1998 ▸, 2000 ▸; Broere & Ruijter, 2012 ▸), cyclo­trimerization of alkynes (Dosa *et al.*, 2002 ▸; Holmes *et al.*, 2015 ▸) and C—S bond activation (Jones & Chin, 1994 ▸; Chan *et al.*, 2015 ▸). The utility of the Cp*Co(CH_2_CH_2_)_2_ complex in organometallic synthesis has been explored extensively. Examples include the preparation of high-oxidation state Co^V^ complexes (Brook­hart *et al.*, 2000 ▸) and the preparation of Cp*Co(η^5^-penta­dien­yl)^+^ complexes (Witherell *et al.*, 2008 ▸; Ylijoki *et al.*, 2009 ▸, 2015 ▸).
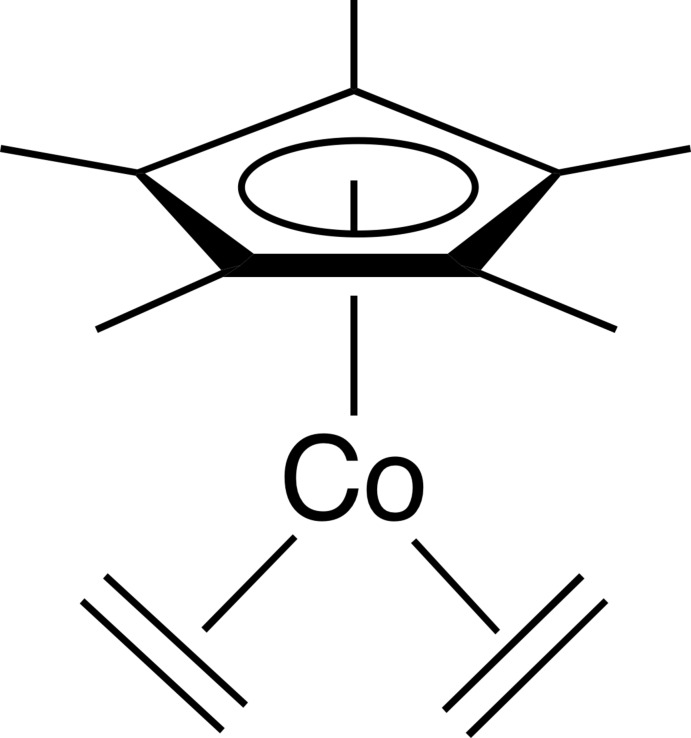


## Structural commentary   

**Figure 1:**
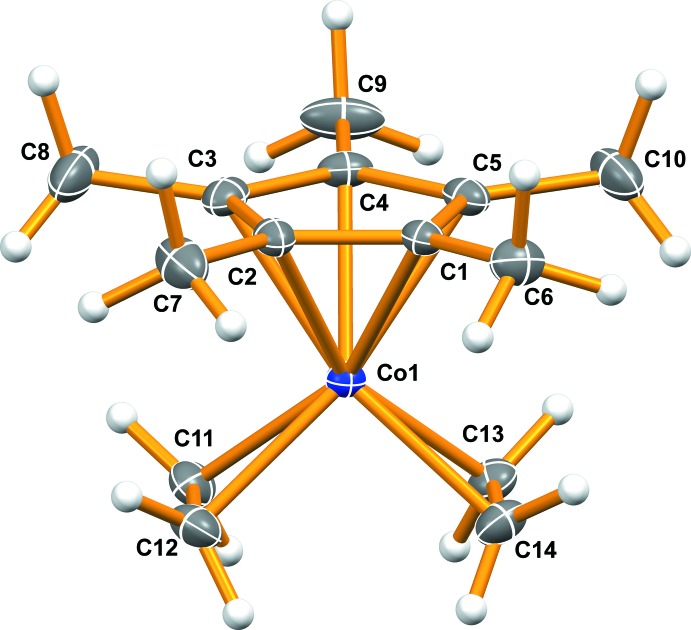
The mol­ecular structure of the title compound, showing the atom labelling. Displacement ellipsoids are drawn at the 50% probability level for non-H atoms.

The mol­ecular structure of the title compound is shown in Fig. 1 ▸. Although the cobalt atom is located on a general position, the mol­ecule is essentially *C*_2v_ symmetric, which agrees with the symmetry of the ^1^H NMR data (Beevor *et al.*, 1981 ▸; Nicholls & Spencer, 1990 ▸). The Co—C(olefin) bonds have an average length of 2.022 (2) Å, while the Co—C(Cp*) bonds average 2.103 (19) Å. The olefin C=C bonds are 1.410 (1) Å. All bond lengths are in agreement with those reported for the related Cp*Cobis(tri­methyl­vinyl­silane) complex (Lenges *et al.*, 1998 ▸). The C11—Co1—C14 and C12—Co1—C13 bond angles average 104.64 (1)°, indicating a parallel arrangement of the olefin ligands. The dihedral angle between the planes defined by the Cp* ligand (C1–C5) and the two olefin ligands (C11–C14) is 0.25 (12)°.

## Supra­molecular features   

**Figure 2:**
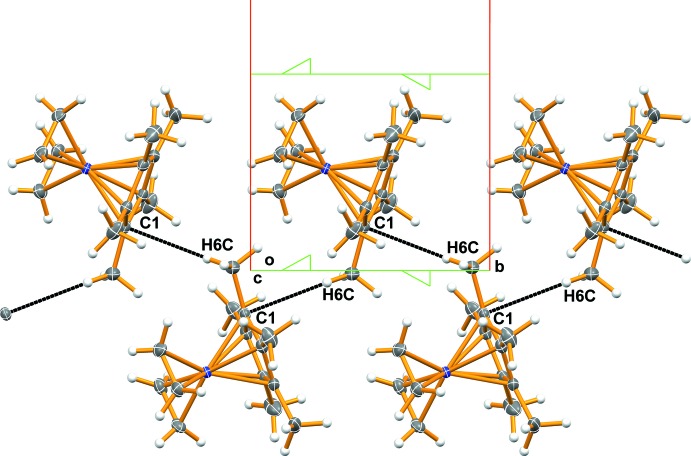
Packing diagram of the title compound, viewed down the *c* axis, showing a chain formed by C—H⋯π inter­actions. Dotted lines show the shortest C—H⋯C contact involved in the inter­action. Ellipsoids are drawn at the 50% probability level. The 2_1_ screw axes (green) are also shown.

In the crystal, a weak C—H⋯π inter­action is observed between one of the methyl groups and the Cp* ring edge of the adjacent mol­ecule related by a 2_1_ screw axis. The shortest contact occurs between the C6—H6*C* of the methyl group and the C1 atom of the Cp* ring [H6*C*⋯C1^i^ 2.79, C6⋯C1^i^ 3.734 (3) Å, C6—H6*C*⋯C1^i^ 162°; symmetry code (i): −*x*, *y* − 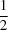, −*z* + 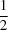] , while the H6*C*⋯Cp* ring centroid distance is 3.00 Å. The mol­ecules are linked through the C—H⋯π inter­actions, forming a helical chain parallel to the *b* axis (Fig. 2 ▸).

## Database survey   

**Figure 3:**
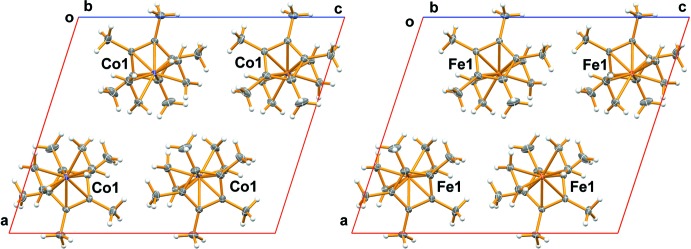
Comparison diagram of the isostructural Cp*Co(CH_2_CH_2_)_2_ (left) and Cp*Fe(CH_2_CH_2_)_2_ (right) unit cells, viewed down the *b* axis.

The Cambridge Structural Database (CSD, Version 5.37; Groom *et al.*, 2016 ▸) contains one additional example of a Cp*Cobis(olefin) complex: the Cp*Cobis(tri­methyl­vinyl­silane) complex (GIQHUJ) reported by Brookhart and co-workers (Lenges *et al.*, 1998 ▸). The title compound is isostructural with the Cp*Fe(CH_2_CH_2_)_2_ complex (VOGJAD; Fig. 3 ▸) reported by Fürstner *et al.* (2008 ▸). The iron compound crystallizes in the monoclinic space group *P*2_1_/*c* with unit-cell dimensions of *a* = 12.5561 (5), *b* = 7.3323 (3), *c* = 14.7157 (6) Å and *β* = 108.3520 (10)° at 100 K.

## Synthesis and crystallization   

The title compound was prepared by reduction of [Co_2_(C_10_H_15_)_2_(μ-Cl)_2_] (Koelle *et al.*, 1986 ▸) under ethyl­ene. This procedure is an adaptation of that reported by Nicholls & Spencer (1990 ▸). All solvents were degassed by purging with nitro­gen and dried by passing through activated Al_2_O_3_. A 1% Na amalgam was prepared by addition of Na (305 mg, 13.3 mmol) in small portions to mercury (30.5 g) in a Schlenk flask equipped with a stir bar and rubber septum under a nitro­gen atmosphere. The sodium was allowed to disperse completely between additions. Gentle heating with a heat gun may be required to initiate the process after the first addition. The Na amalgam was cooled to room temperature. THF (100 ml) was added to the Schlenk flask, followed by gently bubbling ethyl­ene through the system *via* a needle for 20 min to ensure saturation. Previously prepared [Co_2_(C_10_H_15_)(μ-Cl)_2_] (2.77 g, 6.0 mmol) was removed from the glovebox and rapidly added to the Schlenk flask under a nitro­gen purge. The ethyl­ene was bubbled through the THF for an additional 10 min, then the needle was moved to a position *ca* 1 cm above the solution surface to prevent clogging. The reaction was stirred under ethyl­ene for a total of 1.5 h. Over this timespan, the colour evolved from dark brown to a red/orange colour. At this point, the septum was replaced with a glass stopper and the solvent removed completely under vacuum. The evacuated flask was transferred to the glovebox where the product was taken up in pentane and filtered through Celite, taking care to separate the mercury. The solution was concentrated under vacuum in a Schlenk tube and then sealed with a greased glass stopper. The tube was removed from the glovebox and placed in a 193 K freezer overnight. The next day, the tube was removed from the freezer and immediately immersed in a dry ice/acetone bath and placed under inert atmosphere on the Schlenk line. The solvent was removed by canula transfer at low temperature to isolate the title compound (1.8 g, 60%) as dark-red rectangular crystals. The product was dried under vacuum and transferred to the glovebox where it was stored at 233 K. The NMR spectroscopic data is identical to that previously reported (Beevor *et al.*, 1981 ▸; Nicholls & Spencer, 1990 ▸).

## Refinement   

Crystal data, data collection, and structure refinement details are summarized in Table 1 ▸. The H atoms of the methyl groups were included at geometrically idealized positions (C—H = 0.98 Å) and were treated as riding, with *U*_iso_(H) = 1.5*U*_eq_(C). The H atoms of the ethyl­ene groups were located in a difference-Fourier map and their positions were freely refined, while their *U*_iso_(H) values were set to be equal to 1.2*U*_eq_ of the parent carbon atom.

## Supplementary Material

Crystal structure: contains datablock(s) I. DOI: 10.1107/S2056989016012822/is5456sup1.cif

Structure factors: contains datablock(s) I. DOI: 10.1107/S2056989016012822/is5456Isup2.hkl

CCDC reference: 1498272

Additional supporting information: crystallographic information; 3D view; checkCIF report

## Figures and Tables

**Table 1:** Experimental details

Crystal data	
Chemical formula	[Co(C_10_H_15_)(C_2_H_4_)_2_]
*M*_r_	250.25
Crystal system, space group	Monoclinic, *P*2_1_/*c*
Temperature (K)	125
*a*, *b*, *c* (Å)	12.526 (2), 7.2647 (13), 14.712 (3)
β (°)	107.860 (2)
*V* (Å^3^)	1274.3 (4)
*Z*	4
Radiation type	Mo *K*α
μ (mm^−1^)	1.31
Crystal size (mm)	0.23 × 0.12 × 0.10

Data collection	
Diffractometer	Bruker APEXII CCD
Absorption correction	Multi-scan (*SADABS*; Bruker, 2009 ▸)
*T*_min_, *T*_max_	0.524, 0.746
No. of measured, independent and observed [*I* > 2σ(*I*)] reflections	14405, 3165, 2684
*R*_int_	0.084
(sin θ/λ)_max_ (Å^−1^)	0.676

Refinement	
*R*[*F* ^2^ > 2σ(*F* ^2^)], *wR*(*F* ^2^), *S*	0.039, 0.106, 1.06
No. of reflections	3165
No. of parameters	165
H-atom treatment	H atoms treated by a mixture of independent and constrained refinement
Δρ_max_, Δρ_min_ (e Å^−3^)	0.41, −0.51

Computer programs: *APEX2* and *SAINT* (Bruker, 2008 ▸), *SHELXT* (Sheldrick, 2015*a* ▸), *SHELXL2014* (Sheldrick, 2015*b* ▸) and *Mercury* (Macrae *et al.*, 2006 ▸).

## supplementary crystallographic information

### Crystal data

**Table:**

[Co(C_10_H_15_)(C_2_H_4_)_2_]	*F*(000) = 536
*M**_r_* = 250.25	*D*_x_ = 1.304 Mg m^−^^3^
Monoclinic, *P*2_1_/*c*	Mo *K*α radiation, λ = 0.71073 Å
*a* = 12.526 (2) Å	Cell parameters from 7968 reflections
*b* = 7.2647 (13) Å	θ = 2.9–28.7°
*c* = 14.712 (3) Å	µ = 1.31 mm^−^^1^
β = 107.860 (2)°	*T* = 125 K
*V* = 1274.3 (4) Å^3^	Irregular, orange-brown
*Z* = 4	0.23 × 0.12 × 0.10 mm

### Data collection

**Table:**

Bruker APEXII CCD diffractometer	3165 independent reflections
Radiation source: sealed tube	2684 reflections with *I* > 2σ(*I*)
Graphite monochromator	*R*_int_ = 0.084
φ and ω scans	θ_max_ = 28.7°, θ_min_ = 2.9°
Absorption correction: multi-scan (SADABS; Bruker, 2009)	*h* = −16→16
*T*_min_ = 0.524, *T*_max_ = 0.746	*k* = −9→9
14405 measured reflections	*l* = −19→18

### Refinement

**Table:**

Refinement on *F*^2^	0 restraints
Least-squares matrix: full	Hydrogen site location: mixed
*R*[*F*^2^ > 2σ(*F*^2^)] = 0.039	H atoms treated by a mixture of independent and constrained refinement
*wR*(*F*^2^) = 0.106	*w* = 1/[σ^2^(*F*_o_^2^) + (0.0509*P*)^2^ + 0.0513*P*] where *P* = (*F*_o_^2^ + 2*F*_c_^2^)/3
*S* = 1.06	(Δ/σ)_max_ = 0.001
3165 reflections	Δρ_max_ = 0.41 e Å^−^^3^
165 parameters	Δρ_min_ = −0.51 e Å^−^^3^

### Special details

**Table:**

Geometry. All esds (except the esd in the dihedral angle between two l.s. planes) are estimated using the full covariance matrix. The cell esds are taken into account individually in the estimation of esds in distances, angles and torsion angles; correlations between esds in cell parameters are only used when they are defined by crystal symmetry. An approximate (isotropic) treatment of cell esds is used for estimating esds involving l.s. planes.

### Fractional atomic coordinates and isotropic or equivalent isotropic displacement parameters (Å^2^)

**Table:**

	*x*	*y*	*z*	*U*_iso_*/*U*_eq_	
Co1	0.25940 (2)	0.31555 (3)	0.35283 (2)	0.01378 (11)	
C1	0.11064 (16)	0.4768 (2)	0.31455 (13)	0.0162 (4)	
C2	0.15937 (16)	0.4812 (2)	0.23996 (13)	0.0172 (4)	
C3	0.27067 (17)	0.5547 (3)	0.27741 (15)	0.0206 (4)	
C4	0.29067 (17)	0.5974 (3)	0.37588 (15)	0.0204 (4)	
C5	0.19206 (17)	0.5474 (3)	0.39890 (14)	0.0184 (4)	
C6	−0.00806 (17)	0.4243 (3)	0.30531 (16)	0.0247 (4)	
H6A	−0.0577	0.5290	0.2802	0.037*	
H6B	−0.0135	0.3899	0.3681	0.037*	
H6C	−0.0305	0.3197	0.2615	0.037*	
C7	0.10181 (19)	0.4326 (3)	0.13701 (15)	0.0276 (5)	
H7A	0.0436	0.3403	0.1334	0.041*	
H7B	0.1569	0.3826	0.1086	0.041*	
H7C	0.0675	0.5433	0.1020	0.041*	
C8	0.3474 (2)	0.5936 (3)	0.2190 (2)	0.0355 (6)	
H8A	0.3266	0.7112	0.1856	0.053*	
H8B	0.3408	0.4949	0.1722	0.053*	
H8C	0.4250	0.6003	0.2610	0.053*	
C9	0.3941 (2)	0.6847 (3)	0.4412 (2)	0.0350 (6)	
H9A	0.4597	0.6437	0.4237	0.052*	
H9B	0.4027	0.6487	0.5073	0.052*	
H9C	0.3877	0.8189	0.4354	0.052*	
C10	0.1708 (2)	0.5774 (3)	0.49277 (15)	0.0303 (5)	
H10A	0.1248	0.6880	0.4889	0.045*	
H10B	0.2424	0.5931	0.5432	0.045*	
H10C	0.1311	0.4707	0.5075	0.045*	
C11	0.39512 (19)	0.1994 (3)	0.32878 (17)	0.0231 (5)	
H11A	0.436 (2)	0.292 (3)	0.3065 (18)	0.028*	
H11B	0.440 (2)	0.120 (3)	0.3815 (18)	0.028*	
C12	0.29566 (19)	0.1242 (3)	0.26683 (16)	0.0241 (4)	
H12A	0.269 (2)	0.156 (3)	0.201 (2)	0.029*	
H12B	0.273 (2)	0.005 (4)	0.2803 (18)	0.029*	
C13	0.30282 (19)	0.1963 (3)	0.48330 (16)	0.0214 (4)	
H13A	0.370 (2)	0.121 (3)	0.4991 (17)	0.026*	
H13B	0.309 (2)	0.294 (3)	0.5351 (19)	0.026*	
C14	0.20163 (19)	0.1212 (3)	0.42396 (16)	0.0234 (4)	
H14A	0.132 (2)	0.154 (3)	0.4317 (19)	0.028*	
H14B	0.205 (2)	0.008 (4)	0.4038 (18)	0.028*	

### Atomic displacement parameters (Å^2^)

**Table:**

	*U*^11^	*U*^22^	*U*^33^	*U*^12^	*U*^13^	*U*^23^
Co1	0.01325 (17)	0.01235 (16)	0.01530 (17)	0.00046 (8)	0.00374 (11)	0.00026 (9)
C1	0.0174 (9)	0.0125 (8)	0.0188 (9)	0.0025 (7)	0.0059 (8)	0.0006 (7)
C2	0.0205 (9)	0.0151 (9)	0.0163 (9)	0.0042 (7)	0.0060 (7)	0.0018 (7)
C3	0.0200 (10)	0.0164 (9)	0.0272 (10)	0.0046 (7)	0.0100 (8)	0.0071 (8)
C4	0.0178 (9)	0.0124 (9)	0.0275 (10)	0.0001 (7)	0.0021 (8)	0.0011 (7)
C5	0.0215 (10)	0.0147 (9)	0.0176 (9)	0.0042 (7)	0.0041 (8)	−0.0002 (7)
C6	0.0180 (10)	0.0222 (10)	0.0337 (12)	0.0002 (8)	0.0075 (9)	0.0006 (9)
C7	0.0335 (12)	0.0297 (11)	0.0173 (10)	0.0106 (9)	0.0043 (9)	0.0017 (8)
C8	0.0339 (13)	0.0332 (13)	0.0490 (15)	0.0080 (10)	0.0268 (12)	0.0185 (11)
C9	0.0254 (12)	0.0168 (11)	0.0490 (16)	−0.0011 (8)	−0.0088 (11)	−0.0036 (9)
C10	0.0424 (14)	0.0288 (11)	0.0194 (10)	0.0120 (10)	0.0092 (10)	−0.0029 (9)
C11	0.0208 (11)	0.0213 (10)	0.0304 (12)	0.0051 (8)	0.0128 (9)	0.0003 (8)
C12	0.0282 (11)	0.0195 (10)	0.0242 (11)	0.0059 (9)	0.0073 (9)	−0.0038 (8)
C13	0.0237 (11)	0.0191 (10)	0.0201 (10)	0.0024 (8)	0.0047 (9)	0.0053 (7)
C14	0.0223 (11)	0.0200 (10)	0.0279 (11)	−0.0027 (8)	0.0078 (9)	0.0060 (9)

### Geometric parameters (Å, º)

**Table:**

Co1—C14	2.019 (2)	C7—H7A	0.9800
Co1—C13	2.022 (2)	C7—H7B	0.9800
Co1—C12	2.023 (2)	C7—H7C	0.9800
Co1—C11	2.024 (2)	C8—H8A	0.9800
Co1—C5	2.0875 (19)	C8—H8B	0.9800
Co1—C3	2.0894 (19)	C8—H8C	0.9800
Co1—C4	2.093 (2)	C9—H9A	0.9800
Co1—C2	2.1214 (19)	C9—H9B	0.9800
Co1—C1	2.1256 (18)	C9—H9C	0.9800
C1—C2	1.410 (3)	C10—H10A	0.9800
C1—C5	1.438 (3)	C10—H10B	0.9800
C1—C6	1.500 (3)	C10—H10C	0.9800
C2—C3	1.436 (3)	C11—C12	1.409 (3)
C2—C7	1.506 (3)	C11—H11A	0.97 (3)
C3—C4	1.427 (3)	C11—H11B	0.99 (3)
C3—C8	1.500 (3)	C12—H12A	0.95 (3)
C4—C5	1.425 (3)	C12—H12B	0.95 (3)
C4—C9	1.497 (3)	C13—C14	1.410 (3)
C5—C10	1.501 (3)	C13—H13A	0.98 (3)
C6—H6A	0.9800	C13—H13B	1.03 (2)
C6—H6B	0.9800	C14—H14A	0.95 (3)
C6—H6C	0.9800	C14—H14B	0.88 (3)
			
C14—Co1—C13	40.83 (9)	C4—C5—C1	108.86 (17)
C14—Co1—C12	91.64 (10)	C4—C5—C10	126.40 (19)
C13—Co1—C12	104.64 (9)	C1—C5—C10	124.54 (19)
C14—Co1—C11	104.65 (9)	C4—C5—Co1	70.28 (11)
C13—Co1—C11	89.51 (9)	C1—C5—Co1	71.48 (10)
C12—Co1—C11	40.74 (9)	C10—C5—Co1	128.29 (14)
C14—Co1—C5	98.68 (9)	C1—C6—H6A	109.5
C13—Co1—C5	93.17 (8)	C1—C6—H6B	109.5
C12—Co1—C5	161.45 (9)	H6A—C6—H6B	109.5
C11—Co1—C5	146.92 (8)	C1—C6—H6C	109.5
C14—Co1—C3	161.80 (9)	H6A—C6—H6C	109.5
C13—Co1—C3	144.88 (8)	H6B—C6—H6C	109.5
C12—Co1—C3	100.05 (9)	C2—C7—H7A	109.5
C11—Co1—C3	93.25 (8)	C2—C7—H7B	109.5
C5—Co1—C3	66.51 (8)	H7A—C7—H7B	109.5
C14—Co1—C4	132.78 (9)	C2—C7—H7C	109.5
C13—Co1—C4	106.43 (8)	H7A—C7—H7C	109.5
C12—Co1—C4	134.99 (9)	H7B—C7—H7C	109.5
C11—Co1—C4	108.11 (8)	C3—C8—H8A	109.5
C5—Co1—C4	39.85 (8)	C3—C8—H8B	109.5
C3—Co1—C4	39.89 (8)	H8A—C8—H8B	109.5
C14—Co1—C2	125.65 (9)	C3—C8—H8C	109.5
C13—Co1—C2	155.27 (8)	H8A—C8—H8C	109.5
C12—Co1—C2	95.30 (8)	H8B—C8—H8C	109.5
C11—Co1—C2	115.21 (8)	C4—C9—H9A	109.5
C5—Co1—C2	66.16 (7)	C4—C9—H9B	109.5
C3—Co1—C2	39.86 (8)	H9A—C9—H9B	109.5
C4—Co1—C2	66.92 (8)	C4—C9—H9C	109.5
C14—Co1—C1	95.63 (8)	H9A—C9—H9C	109.5
C13—Co1—C1	116.50 (8)	H9B—C9—H9C	109.5
C12—Co1—C1	124.04 (8)	C5—C10—H10A	109.5
C11—Co1—C1	153.97 (8)	C5—C10—H10B	109.5
C5—Co1—C1	39.89 (7)	H10A—C10—H10B	109.5
C3—Co1—C1	66.27 (7)	C5—C10—H10C	109.5
C4—Co1—C1	66.98 (7)	H10A—C10—H10C	109.5
C2—Co1—C1	38.79 (7)	H10B—C10—H10C	109.5
C2—C1—C5	107.56 (17)	C12—C11—Co1	69.58 (12)
C2—C1—C6	126.07 (18)	C12—C11—H11A	120.6 (16)
C5—C1—C6	126.15 (17)	Co1—C11—H11A	109.4 (15)
C2—C1—Co1	70.45 (11)	C12—C11—H11B	117.1 (15)
C5—C1—Co1	68.63 (10)	Co1—C11—H11B	114.1 (14)
C6—C1—Co1	130.39 (13)	H11A—C11—H11B	116 (2)
C1—C2—C3	108.12 (17)	C11—C12—Co1	69.67 (12)
C1—C2—C7	126.10 (18)	C11—C12—H12A	122.1 (16)
C3—C2—C7	125.60 (18)	Co1—C12—H12A	113.1 (15)
C1—C2—Co1	70.77 (11)	C11—C12—H12B	118.7 (15)
C3—C2—Co1	68.87 (11)	Co1—C12—H12B	110.9 (15)
C7—C2—Co1	129.77 (13)	H12A—C12—H12B	113 (2)
C4—C3—C2	108.54 (17)	C14—C13—Co1	69.45 (12)
C4—C3—C8	126.7 (2)	C14—C13—H13A	118.6 (15)
C2—C3—C8	124.6 (2)	Co1—C13—H13A	115.4 (14)
C4—C3—Co1	70.19 (11)	C14—C13—H13B	124.8 (15)
C2—C3—Co1	71.27 (11)	Co1—C13—H13B	110.3 (13)
C8—C3—Co1	128.37 (15)	H13A—C13—H13B	111 (2)
C5—C4—C3	106.90 (17)	C13—C14—Co1	69.72 (12)
C5—C4—C9	126.8 (2)	C13—C14—H14A	121.0 (16)
C3—C4—C9	126.3 (2)	Co1—C14—H14A	112.1 (15)
C5—C4—Co1	69.87 (11)	C13—C14—H14B	116.1 (16)
C3—C4—Co1	69.92 (11)	Co1—C14—H14B	114.5 (17)
C9—C4—Co1	126.91 (14)	H14A—C14—H14B	115 (2)
			
C5—C1—C2—C3	−0.1 (2)	C8—C3—C4—C9	−2.0 (3)
C6—C1—C2—C3	174.76 (17)	Co1—C3—C4—C9	121.7 (2)
Co1—C1—C2—C3	−58.94 (13)	C2—C3—C4—Co1	61.26 (13)
C5—C1—C2—C7	−175.46 (17)	C8—C3—C4—Co1	−123.7 (2)
C6—C1—C2—C7	−0.5 (3)	C3—C4—C5—C1	−1.0 (2)
Co1—C1—C2—C7	125.75 (19)	C9—C4—C5—C1	176.98 (18)
C5—C1—C2—Co1	58.80 (12)	Co1—C4—C5—C1	−61.39 (13)
C6—C1—C2—Co1	−126.29 (18)	C3—C4—C5—C10	−175.97 (18)
C1—C2—C3—C4	−0.4 (2)	C9—C4—C5—C10	2.0 (3)
C7—C2—C3—C4	174.89 (18)	Co1—C4—C5—C10	123.6 (2)
Co1—C2—C3—C4	−60.58 (13)	C3—C4—C5—Co1	60.42 (13)
C1—C2—C3—C8	−175.64 (18)	C9—C4—C5—Co1	−121.6 (2)
C7—C2—C3—C8	−0.3 (3)	C2—C1—C5—C4	0.7 (2)
Co1—C2—C3—C8	124.2 (2)	C6—C1—C5—C4	−174.21 (18)
C1—C2—C3—Co1	60.13 (13)	Co1—C1—C5—C4	60.64 (13)
C7—C2—C3—Co1	−124.53 (19)	C2—C1—C5—C10	175.81 (18)
C2—C3—C4—C5	0.9 (2)	C6—C1—C5—C10	0.9 (3)
C8—C3—C4—C5	175.94 (19)	Co1—C1—C5—C10	−124.24 (19)
Co1—C3—C4—C5	−60.39 (13)	C2—C1—C5—Co1	−59.94 (13)
C2—C3—C4—C9	−177.08 (18)	C6—C1—C5—Co1	125.15 (18)

## References

[bb1] Beevor, R. G., Frith, S. A. & Spencer, J. L. (1981). *J. Organomet. Chem.* **221**, C25–C27.

[bb2] Broere, D. L. J. & Ruijter, E. (2012). *Synthesis*, **44**, 2639–2672.

[bb3] Brookhart, M., Grant, B. E., Lenges, C. P., Prosenc, M. H. & White, P. S. (2000). *Angew. Chem. Int. Ed.* **39**, 1676–1679. 10.1002/(sici)1521-3773(20000502)39:9<1676::aid-anie1676>3.0.co;2-m 10820474

[bb4] Bruker (2008). *APEX2* and *SAINT*. Bruker AXS Inc., Madison, Wisconsin, USA.

[bb5] Bruker (2009). *SADABS*. Bruker AXS Inc., Madison, Wisconsin, USA.

[bb6] Chan, N. H., Roache, J. H. & Jones, W. D. (2015). *Inorg. Chim. Acta*, **437**, 36–40.

[bb7] Dosa, P. I., Whitener, G. D., Vollhardt, K. P. C., Bond, A. D. & Teat, S. J. (2002). *Org. Lett.* **4**, 2075–2078. 10.1021/ol025956o 12049521

[bb8] Fürstner, A., Martin, R., Krause, H., Seidel, G., Goddard, R. & Lehmann, C. W. (2008). *J. Am. Chem. Soc.* **130**, 8773–8787. 10.1021/ja801466t 18597432

[bb9] Groom, C. R., Bruno, I. J., Lightfoot, M. P. & Ward, S. C. (2016). *Acta Cryst.* B**72**, 171–179. 10.1107/S2052520616003954 PMC4822653 27048719

[bb10] Holmes, D., Lee, S. Y., Lotz, S. D., Nguyen, S., Schaller, G. R., Schmidt-Radde, R. H. & Vollhardt, K. P. C. (2015). *Synthesis*, **47**, 2038–2054.

[bb11] Jones, W. D. & Chin, R. M. (1994). *J. Organomet. Chem.* **472**, 311–316.

[bb12] Koelle, U., Fuss, B., Belting, M. & Raabe, E. (1986). *Organometallics*, **5**, 980–987.

[bb13] Lenges, C. P., Brookhart, M. & Grant, B. E. (1997). *J. Organomet. Chem.* **528**, 199–203.

[bb14] Lenges, C. P., White, P. S. & Brookhart, M. (1998). *J. Am. Chem. Soc.* **120**, 6965–6979.

[bb15] Lenges, C. P., White, P. S., Marshall, W. J. & Brookhart, M. (2000). *Organometallics*, **19**, 1247–1254.

[bb16] Macrae, C. F., Edgington, P. R., McCabe, P., Pidcock, E., Shields, G. P., Taylor, R., Towler, M. & van de Streek, J. (2006). *J. Appl. Cryst.* **39**, 453–457.

[bb17] Nicholls, J. C. & Spencer, J. L. (1990). *Inorg. Synth.* **28**, 278–280.

[bb18] Sheldrick, G. M. (2015*a*). *Acta Cryst.* A**71**, 3–8.

[bb19] Sheldrick, G. M. (2015*b*). *Acta Cryst.* C**71**, 3–8.

[bb20] Witherell, R. D., Ylijoki, K. E. O. & Stryker, J. M. (2008). *J. Am. Chem. Soc.* **130**, 2176–2177. 10.1021/ja710568d 18225907

[bb21] Ylijoki, K. E. O., Kirk, A. D., Böcklein, S., Witherell, R. D. & Stryker, J. M. (2015). *Organometallics*, **34**, 3335–3357.

[bb22] Ylijoki, K. E. O., Witherell, R. D., Kirk, A. D., Böcklein, S., Lofstrand, V. A., McDonald, R., Ferguson, M. J. & Stryker, J. M. (2009). *Organometallics*, **28**, 6807–6822.

